# Mild Gait Impairment and Its Potential Diagnostic Value in Patients with Early-Stage Parkinson's Disease

**DOI:** 10.1155/2021/6696454

**Published:** 2021-04-02

**Authors:** Zhuang Wu, Xu Jiang, Min Zhong, Bo Shen, Jun Zhu, Yang Pan, Jingde Dong, Pingyi Xu, Wenbin Zhang, Jun Yan, Li Zhang

**Affiliations:** ^1^Department of Geriatric Neurology, Affiliated Brain Hospital of Nanjing Medical University, Nanjing, China; ^2^Department of Neurology, First Affiliated Hospital of Guangzhou Medical University, Guangzhou, China; ^3^Department of Neurosurgery, Affiliated Brain Hospital of Nanjing Medical University, Nanjing, China

## Abstract

**Methods:**

32 patients with early-stage PD and 30 healthy control subjects (HC) were enrolled. All participants completed the instrumented stand and walk test, and gait data was collected using wearable sensors.

**Results:**

We observed increased variability of stride length (SL) (*P* < 0.001), stance phase time (StPT) (*P* = 0.004), and swing phase time (SwPT) (*P* = 0.011) in PD. There were decreased heel strike (HS) (*P* = 0.001), range of motion of knee (*P* = 0.036), and hip joints (*P* < 0.001) in PD. In symmetry analysis, no difference was found in any of the assessed gait parameters between HC and PD. Only total steps (AUC = 0.763, *P* < 0.001), SL (AUC = 0.701, *P* = 0.007), SL variability (AUC = 0.769, *P* < 0.001), StPT variability (AUC = 0.712, *P* = 0.004), and SwPT variability (AUC = 0.688, *P* = 0.011) had potential diagnostic value. When these five gait parameters were combined, the predictive power was found to increase, with the highest AUC of 0.802 (*P* < 0.001).

**Conclusions:**

Patients with early-stage PD presented increased variability but still symmetrical gait pattern. Some specific gait parameters can be applied to diagnose early-stage PD which may increase diagnosis accuracy. Our findings are helpful to improve patient's quality of life.

## 1. Introduction

Gait damage is a common feature in patients with Parkinson's disease (PD). The gait characteristics of PD are decreased pace, step length, and arm swing [1–3]. As the disease progresses, some patients may suffer from the festination and freezing of gait [4, 5]. These disorders may induce falls and fractures, which increase mortality [6]. Thus, in the gait analysis of PD, identifying changes in the gait characteristics is a priority. With the rapid development of technology, wearable sensors can be used to quantify gait parameters. However, few studies about quantitative gait analysis in early-stage PD have been published. In early-stage PD, affected individuals walk at a slower pace and with a more variable and asymmetric gait pattern than normal [7]. Foot heights during heel strike are significantly decreased which are reflective of dragging the foot in early-stage PD [8]. A reduction in physical activity and gait speed is also associated with prodromal PD [9]. During a long-term follow-up of patients with early-stage PD, stride length and step time variability increased when patients walked at a normal pace [10]. All these studies suggest that patients with early-stage PD already have gait damage. However, gait characteristics extensively vary with no consistency across studies [7, 11]. Previous studies also have some limitations. First, most of them have focused on spatiotemporal gait parameters. These spatiotemporal gait parameters distantly reflect the gait changes of patients with PD as they lack disease specificity [12–14]. Clinical gait analysis mainly includes spatiotemporal and kinematic gait parameters [15]. Further studies validating changes in kinematic gait parameters in early-stage PD are needed. Second, appropriate technological solutions, such as wearable sensors, can improve PD diagnosis [16]. For example, previous studies have demonstrated that postural control is compromised in early-stage PD and may thus act as a diagnostic biomarker [17, 18]. However, similar articles are rare, and only five papers dealing the early diagnosis have been summarized in a recent review [19]. Accordingly, the present study is aimed at (1) comparing the differences in spatiotemporal gait parameters, kinematic gait parameters, and variability and symmetry analyses of gait performance between patients with early-stage PD and normal people and (2) evaluating the diagnostic value of gait parameters for patients with early-stage PD. We hypothesized that patients with early-stage PD present an asymmetrical and variable gait pattern. Some spatiotemporal and kinematic gait parameters can be applied to diagnose early-stage PD. Our results may aid the diagnosis of early-stage PD. Early identification of gait damage in patients with PD is beneficial to the choice of treatment methods such as drugs and rehabilitation. Targeted improvement of the patient's gait will help improve the patient's quality of life.

## 2. Methods

### 2.1. Participants

A total of 32 patients with early-stage PD (22 men, and 10 women; mean duration of disease 2.41 ± 1.30 years) were recruited from the Department of Geriatrics, Affiliated Brain Hospital of Nanjing Medical University, between October 2018 and November 2019. We also recruited 30 HC from the caregivers of the patients with PD. Inclusion criteria for early-stage PD were as follows: (1) diagnosis of PD according to the Movement Disorder Society (MDS) criteria [20], (2) Hoehn-Yahr (H-Y) stage of 1-2, and (3) disease duration of <4 years [21]. Exclusion criteria for PD were as follows: (1) other diseases that could affect gait, including cerebrovascular disease, orthopedic disease, and spinal column diseases; (2) inability to follow doctor's instructions; (3) have received other PD therapies, i.e., rehabilitation therapy. Inclusion criteria for HC were as follows: (1) no medical history of PD, cerebrovascular disease, orthopedic disease, and spinal column diseases; (2) ability to follow doctor's instructions. Ethical approval was obtained from the Medical Ethics Committee of the Affiliated Brain Hospital of Nanjing Medical University. After a complete explanation of the study to all participants, they signed a written informed consent before the experiment. All above-mentioned procedures were performed according to the declaration of Helsinki.

### 2.2. Demographic and Clinical Measures

For all participants, we collected the following demographic characteristics: age, height, weight, gender, and degree of education. Cognition was assessed with the Montreal Cognitive Assessment (MoCA). All participants were tested in the morning. The Unified Parkinson's Disease Rating Scale (UPDRS) and H-Y scale were used to assess the severity of PD motor symptoms. For patients with PD, their antiparkinsonian medication was stopped for at least 24 h (72 h for controlled-release antiparkinsonian medication).

### 2.3. Quantitative Gait Evaluation

All participants completed the instrumented stand and walk test (ISAW), a reliable and sensitive method of measuring gait [22]. All participants were asked to stand quietly for 30 seconds with their arms at their sides and look straight ahead, then walking 7 meters at a self-select and comfortable speed, turning 180° and returned to their initial place. We explained the steps of ISAW in detail to all participants before the test. Also, all participants walked twice in advance to be familiar with the test. After that, we started to collect gait data. When all subjects underwent this test, gait data was collected at the same time.

### 2.4. Equipment

We used the JiBuEn gait-analysis system to collect gait data. This gait-analysis system comprised shoes and modules with Micro-Electro-Mechanical System sensors fixed behind the upper and lower limbs, under the shoe heel bottom. The system gathered motion information and transmitted it to a computer. The hexahedral calibration technique, high-order low-pass filter, and zero-correction algorithm were used in data preprocessing. The accuracy of this system has been tested before [23]. Through the latest JiBuEn gait-analysis system, we can obtain spatiotemporal gait parameters (total steps of ISAW, stride length, gait velocity, cadence, stride time, stance phase time, swing phase time, variability of stride length, variability of stride time, variability of stance phase time, and variability of swing phase time) and kinematic gait parameters (heel strike angle, toe-off angle, and range of motion of ankle, knee, and hip joints).

### 2.5. Statistical Analysis

Data are expressed as the mean ± standard deviation. The significance level was set at 0.05. Count data were given as percentages. For both groups, measurement data were initially analyzed with the Kolmogorov-Smirnov test. For normally distributed data, the independent *t*-test was used to perform intergroup comparison of measurement data. For nonnormally distributed data of intergroup gait characteristics, the Mann-Whitney *U* Test was used. The *χ*^2^ test was used for count data. Variability of gait parameters from the left and right sides were calculated separately (Equation (1)) and then combined (Equation (2)). This method can avoid confusion due to step changes caused by the asymmetry between the left and right sides in PD [24]. The symmetry of gait parameters was assessed through the asymmetry index (AI) (Equation (3)) [25–27].

(1) $$\begin{array}{c} {CV}_{separate}=standard\;deviation÷mean, \end{array}$$

(2) $$\begin{array}{c} \%{CV}_{combined}=\sqrt{\frac{{CV}_{L}+CV_{R}}{2}}*100. \end{array}$$

The subscripts *R* and *L* represent the right and left sides of participants, respectively. CV means coefficient of variation.

(3) $$\begin{array}{c} \%AI=\frac{\;\max\;\left(X_{L},X_{R}\right)-\min\;\left(X_{L},X_{R}\right)}{\max\;\left(X_{L},X_{R}\right)}*100, \end{array}$$

where *X* = [SL, ST, StPT, SwPT, HS, TO, ROM − AJ, ROM − KJ, ROM − HJ], the subscripts *R* and *L* represent the right and left sides of participants, respectively. SL: stride length; ST: stride time; StPT: stance phase time; SwPT: swing phase time; HS: heel strike angle; TO: toe-off angle; ROM: range of motion; AJ: ankle joint; KJ: knee joint; and HJ: hip joint.

The predictive performance of gait parameters was evaluated by receiver operating characteristic (ROC) curve analysis. The logistic regression model was used to evaluate different predictive parameters and calculate predictive probability. Predictive probability was then used for ROC analysis. The optimum cut-off values to predict PD were calculated with Youden Index. IBM SPSS software version 23 was used for data analyses. Figures were configured using Graph Pad Prism Software version 8.0.1.

## 3. Results

### 3.1. Clinical Characteristics of Participants

Sixty-two participants were included in this study, and their demographic, cognitive, and clinical characteristics are shown in Table 1. Among the 32 PD patients, 22 (68.8%) were male and 20 (62.5%) started with the left side, the mean age was 65.66 ± 10.16 years, the mean height was 165.78 ± 6.74 cm, and the mean weight was 65.94 ± 10.05 kg. Moreover, 4 (12.5%), 7(21.9%), 17 (53.1%), and 4 (12.5%) cases received education of illiteracy, primary school, middle school, and college, respectively. The mean duration of PD was 2.41 ± 1.30 years, and the mean Hoehn-Yahr (H-Y) stage of the disease was 1.73 ± 0.44. The total UPDRS III score was 22.91 ± 9.32.

### 3.2. Changes in Spatiotemporal Gait Parameters

We measured spatiotemporal gait parameters, including total steps (TS) of ISAW, stride length (SL), gait velocity (GV), cadence (CA), stride time (ST), stance phase time (StPT), and swing phase time (SwPT). Moreover, we calculated the variabilities of SL (CV-SL), ST (CV-ST), StPT (CV-StPT), and SwPT (CV-SwPT). We observed only slight differences between the HC and early-stage PD in these spatiotemporal gait parameters (Table 2). For patients with early-stage PD, the TS of ISAW was 12.81 ± 3.42 steps, which was a significant increase of ~24.01% compared with that of the HC. Compared with the HC, SL decreased by ~9.32% in early-stage PD. We also observed increased variability of SL (*P* < 0.001), StPT (*P* = 0.004), and SwPT (*P* = 0.011) in early-stage PD.

### 3.3. Changes in Kinematic Gait Parameters

**Figure 1:**
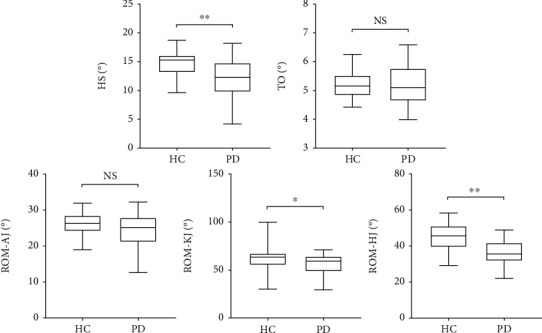
Changes in kinematic gait parameters. HS: heel strike angle; TO: toe-off angle; ROM: range of motion; AJ: ankle joint; KJ: knee joint; HJ: hip joint. “ns” means no significance, ^∗^*P* < 0.05, ^∗∗^*P* ≤ 0.001.

Kinematic gait parameters were evaluated based on the range of motion (ROM) of the ankle, knee, and hip joints. ROM was defined as the difference between the minimum and maximum angles of the above three joints in the sagittal plane. Moreover, toe-off (TO) and heel strike (HS) angles were included in our study (Figure 1). We observed significant differences in HS, ROM-knee joints (ROM-KJ), and ROM-hip joints (ROM-HJ) between two groups but none in TO and ROM-ankle joints (ROM-AJ) between two groups.

### 3.4. Symmetry Analysis of Gait Parameters

We included the spatiotemporal and kinematic gait parameters in the symmetry analysis. In the analysis of gait symmetry, there was no difference in any of the assessed gait parameters between the HC and PD (Table 3).

### 3.5. ROC Analysis of Gait Parameters

**Figure 2:**
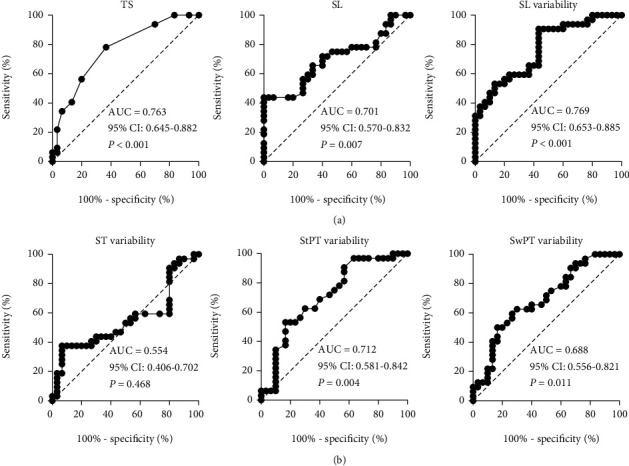
Receiver operating characteristics (ROC) analysis for gait parameters. TS: total steps; SL: stride length; SL variability: stride length variability; ST variability: stride time variability; StPT variability: stance phase time variability; SwPT variability: swing phase time variability; AUC: area under the curve; CI: confidence interval.

We used the ROC curve to evaluate the value of gait parameters in predicting early-stage PD to HC. We found only a few gait parameters with potential diagnostic value (Figure 2). TS, SL, and SL variability showed significant value in predicting early-stage PD with AUCs of 0.763 (95%CI = 0.645 − 0.882; *P* < 0.001), 0.701 (95%CI = 0.570 − 0.832; *P* = 0.007), and 0.769 (95%CI = 0.653 − 0.885; *P* < 0.001), respectively. At a cut-off of 10 steps, TS offered the best accuracy in predicting early-stage PD with the sensitivity and specificity of 78.12% and 63.33%, respectively. 1.045 was the optimum cut-off of SL. The sensitivity and specificity were 43.75% and 100%, respectively. With the cut-off value at 20.820 of SL variability, the sensitivity and specificity were 90.62% and 56.67%, respectively. No significant value for ST variability was observed in predicting early-stage PD with an AUC of 0.554 (95%CI = 0.406–0.702; *P* = 0.468). However, either StPT variability or SwPT variability can effectively predict early-stage PD, with AUCs of 0.712 (95%CI = 0.581–0.842; *P* = 0.004) and 0.688 (95%CI = 0.556–0.821; *P* = 0.011), respectively. The optimum cut-off of StPT variability was 16.125, clearly distinguishing between early-stage PD and HC. Sensitivity and specificity were 53.13% and 83.33%, respectively. At a cut-off of 21.794, SwPT variability offered the highest accuracy in predicting PD with sensitivity and specificity of 50.00% and 83.33%, respectively. We further explored the predictive value of kinematic gait parameters and found that HS, TO, ROM-AJ, ROM-KJ, and ROM-HJ all cannot predict early-stage PD (figures not shown in this article).

**Figure 3:**
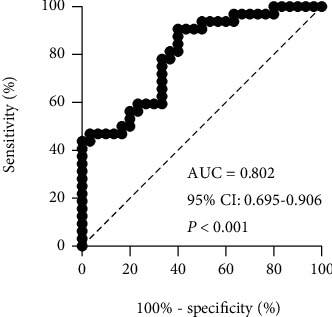
Receiver operating characteristics (ROC) analysis for the combination of TS, SL, SL variability, StPT variability, and SwPT variability. The combination of those five gait parameters increased the predictive power with the highest AUC of 0.802 (95% CI 0.695–0.906, *P* < 0.001). TS: total steps; SL: stride length; SL variability: stride length variability; StPT variability: stance phase time variability; SwPT variability: swing phase time variability; AUC: area under the curve; CI: confidence interval.

To explore the predictive value of the combination of TS, SL, SL variability, StPT variability, and SwPT variability, we combined these five gait parameters in a logistical analysis model to calculate probability. We then used ROC analysis to calculate the AUC (Figure 3). When the five gait parameters were combined, the predictive power was found to increase, with the highest AUC of 0.802 (95%CI = 0.695–0.906; *P* < 0.001). At a cut-off value of 0.388, the sensitivity and specificity of the association to predict early-stage PD were 90.62% and 60.00%, respectively.

## 4. Discussion

This study was a cross-sectional, single-center, observational one that was conducted to (1) quantify gait impairments in early-stage PD using wearable sensors from spatiotemporal gait parameters, kinematic gait parameters, and variability and symmetry analyses of gait parameters and (2) evaluate the predictive value of gait parameters for early-stage PD. Our finding may aid the diagnosis of early-stage PD and improve personalized care in patients with early-stage PD.

A previous study has demonstrated that SL was the most prominent parameter of altered gait in initial stages of PD patients [28]. During a long-term follow-up of patients with early-stage PD, SL and ST variability increased when patients walked at a normal pace [10]. These passages were consistent with our study. For spatiotemporal parameters, we found impairment only in the TS of ISAW, SL, SL variability, StPT, and SwPT variability in patients with early-stage PD. This finding suggested that gait impairment in patients with PD stems from a short SL and a more variable gait pattern. This is noteworthy because previous research has demonstrated that variability in gait predicted falls in older adults and PD [29]. Although patients with early-stage PD have minor gait impairments, their potential risk of falling cannot be ignored. Another study has used mean step length and mean step length variability to accurately classify PD [7]. Based on previous research and the present one, we used ROC curves to evaluate the predictive value of gait parameters for early-stage PD. We found that TS, SL, SL variability, StPT variability, and SwPT variability can predict PD alone. However, we did not find statistically significant values in other gait parameters predicting PD. Particularly for TS and SL variability, the AUC of these two parameters can reach 0.763 and 0.769, respectively, suggesting that these two parameters had relatively high predictive value. Moreover, when TS, SL, SL variability, StPT variability, and SwPT variability were combined, the predictive power increased and showed the highest AUC of 0.802. This finding is important because diagnosing early-stage PD is a clinical challenge. Overall, our study demonstrated the feasibility of applying gait parameters to diagnose early-stage PD. Quantifying gait parameters using wearable devices, combined with the patient's clinical performance and auxiliary examination, can increase diagnosis accuracy.

The onset of PD is mostly unilateral and it may be attributed to the degeneration of dopaminergic cells starting with an asymmetrical pattern. The consistency of activities on both lower limbs is defined as symmetry [30]. In our study, PD patients with an onset of left and right sides accounted for 62.5% and 37.5%, respectively. We did not find statistical differences in the symmetry analysis of gait parameters. This is inconsistent with our hypothesis and the clinical performance of patients with PD. Previous studies have demonstrated that patients with PD walked in a more asymmetric gait pattern compared to HC [3, 7, 26, 27]. We tentatively attribute this to more advanced stage patients with PD were enrolled in previous studies. These studies all have included patients with H-Y stage 3. It means that the presence of postural instability in some patients of previous studies [31]. However, only patients with H-Y stage 1-2 were included in our research. In addition, the loss of dopamine markers occurred rapidly and virtually completed by 4-year disease duration [21]. Therefore, the disease duration of all patients with PD in our study was less than 4 years. To our best knowledge, this inclusion criterion was not admitted to any previous studies. A study involving patients with H-Y stage 1-1.5 and a mean disease duration of 1.38 years has shown that the gait variables are significantly altered but gait symmetry remains preserved during early-stage PD [28]. Last but not least, in our study, gait speed of HC and early-stage PD were 0.91 ± 0.14 m/s and 0.85 ± 0.20 m/s, respectively. There was no difference in gait speed performance between two groups. This is noteworthy, because many gait parameters are speed-dependent [27]. The comparisons between the other gait parameters can be biased by the different gait speed. This may result in different conclusions because of previous studies failing to control speed. Our study showed that patients with a mean H-Y stage of 1.73 and a mean disease duration of 2.41 years retained their symmetrical gait pattern. A symmetrical gait pattern in patients with early-stage PD might be attributed to that the preserved symmetric gait function in both the motor cortex and supplementary motor cortex which may compensate for an asymmetrical dopaminergic cells distributed pattern in basal ganglia [28]. Based on these studies and our results, we hypothesized that although the onset of PD was mostly unilateral, the gait pattern of early-stage PD remained symmetrical. We found that up to a mean H-Y stage of 1.73, patients with PD retained a symmetrical gait pattern. As the disease progressed, an asymmetrical gait pattern gradually appeared. However, further study is needed to verify our hypothesis.

Previous studies have rarely analyzed kinematic parameters in early-stage PD. We observed significant differences in HS, ROM-KJ, and ROM-HJ between two groups. A smaller HS angle indicated a decreased foot height which reflected foot dragging. A study has demonstrated decreased foot height in early-stage PD [8] which is consistent with our study. Patients with PD showed reduced ROM-AJ, ROM-KJ, and ROM-HJ on both sides [27]. This finding slightly differs from ours because no impairment of ROM-AJ was found in early-stage PD. We attribute the discrepancy to the different methods of calculation. Our ROM calculation method was based on the average value of the left and right sides, and the aforementioned study has calculated them independently which may magnify the difference. Moreover, they have also included patients with H-Y stage 3. From the distribution of damaged joints, we speculated that gait damage started from the proximal joints and affected the distal joint as the disease progressed. Our research extended previous findings in showing that the gait damage of patients with early-stage PD was mild and primarily focused on kinematic parameters. However, it is the spatiotemporal gait parameters that had potential value for early PD diagnosis.

The strengths of our study are as follows. First, gait impairments in early-stage PD were comprehensively analyzed by using wearable sensors from spatiotemporal gait parameters, kinematic gait parameters, and variability and symmetry analyses of gait parameters. Second, we extended previous studies by exploring the predictive value of gait parameters in early-stage PD. Third, the disease duration of all patients with PD in our study was less than 4 years. This inclusion criterion was not admitted to previous studies since the loss of dopamine markers occurred rapidly and virtually completed by 4 years disease duration. However, our study also has several limitations. First, our study had a small sample size and was conducted at a single center. Therefore, clinical inspection can have a selection bias. Second, this was not a de novo group. Some of these patients had already taken anti-PD drugs. However, their antiparkinsonian medication was stopped for at least 24 h (72 h for controlled-release antiparkinsonian medication) to minimize the impact of drugs on gait performance. Third, PD is a kind of heterogeneous disease, and the possible influence of nonmotor symptoms on gait performance was not accounted for in our study.

## 5. Conclusion

In conclusion, gait damage of patients with early-stage PD was mild and mostly focuses on kinematic gait parameters. Patients with early-stage PD presented increased variability but still symmetrical gait pattern. Some spatiotemporal gait parameters, e.g., TS of ISAW, SL, SL variability, StPT, and SwPT variability, can be applied to help diagnose early-stage PD. Quantifying gait parameters using wearable devices, combined with the patient's clinical performance and auxiliary examination, may increase diagnosis accuracy. Our findings are helpful to reveal gait impairments in patients with early-stage PD. Choosing the corresponding treatment methods based on the revealed gait damage is essential to improve the patient's quality of life. Further research, especially longitudinal cohort and de novo group ones, is needed to evaluate the evolution of PD gait pattern. This is a dynamic process.

## Figures and Tables

**Table 1:** Clinical characteristics of participants.

	HC	PD	*P*
*N*	30	32	
Age (years)	62.43 ± 6.43	65.66 ± 10.16	0.139
Height (cm)	163.23 ± 4.72	165.78 ± 6.74	0.106
Weight (kg)	63.23 ± 8.61	65.94 ± 10.05	0.261
Male (%)	17 (56.7)	22 (68.8)	0.325
MoCA	25.40 ± 1.16	25.00 ± 2.58	0.618
Education (%)			0.094
Illiteracy	1 (3.3)	4 (12.5)	
Primary school	7 (23.4)	7 (21.9)	
Middle school	22 (73.3)	17 (53.1)	
College	0	4 (12.5)	
Duration of PD (years)		2.41 ± 1.30	
H-Y stage		1.73 ± 0.44	
Onset side (%)		Left (62.5)	
UPDRS III total scores		22.91 ± 9.32	

Data is shown as Mean ± SD. MoCA: Montreal Cognitive Assessment; H-Y stage: Hoehn-Yahr stage; UPDRS III: Unified Parkinson's Disease Rating Scale part 3.

**Table 2:** Spatiotemporal gait parameters of participants.

	HC	PD	*P*
TS (steps)	10.33 ± 2.09	12.81 ± 3.42	0.001^∗∗^
SL (m)	1.18 ± 0.10	1.07 ± 0.16	0.003^∗^
GV (m/s)	0.91 ± 0.14	0.85 ± 0.20	0.204
CA (steps/min)	93.27 ± 9.21	95.20 ± 16.99	0.576
ST (s)	1.30 ± 0.14	1.31 ± 0.28	0.899
StPT (%)	64.88 ± 2.21	64.75 ± 5.22	0.312
SwPT (%)	35.12 ± 2.21	35.25 ± 5.22	0.396
CV-SL (%)	20.92 ± 2.82	24.88 ± 4.65	<0.001^∗∗^
CV-ST (%)	21.38 ± 5.10	23.25 ± 6.90	0.229
CV-StPT (%)	14.80 ± 3.07	17.23 ± 4.56	0.004^∗^
CV-SwPT (%)	19.67 ± 4.57	23.14 ± 6.80	0.011^∗^

Data is shown as Mean ± SD. TS: total steps; SL: stride length; GV: gait velocity; CA: cadence; ST: stride time; StPT: stance phase time; SwPT: swing phase time; CV: coefficient of variation; ^∗^*P* < 0.05; ^∗∗^*P* ≤ 0.001.

**Table 3:** Symmetry analysis of gait parameters.

	HC	PD	*P*
AI-SL (%)	2.47 ± 0.88	2.27 ± 0.86	0.406
AI-ST (%)	6.64 ± 8.43	9.99 ± 10.76	0.083
AI-StPT (%)	4.75 ± 5.80	5.53 ± 5.47	0.434
AI-SwPT (%)	8.09 ± 8.71	9.56 ± 8.49	0.451
AI-HS (%)	16.41 ± 8.83	19.84 ± 13.70	0.250
AI-TO (%)	9.75 ± 6.19	10.92 ± 9.01	0.554
AI-ROM-AJ (%)	9.83 ± 7.36	12.11 ± 10.84	0.693
AI-ROM-KJ (%)	15.02 ± 15.16	16.92 ± 15.80	0.612
AI-ROM-HJ (%)	7.04 ± 5.44	12.61 ± 11.62	0.061

Data is shown as Mean ± SD. AI: asymmetry index; SL: stride length; ST: stride time; StPT: stance phase time; SwPT: swing phase time; HS: heel strike angle; TO: toe-off angle; ROM: range of motion; AJ: ankle joint; KJ: knee joint; HJ: hip joint.

## Data Availability

The data used to support the findings of this study are available from the corresponding author upon request.
